# Total *Panax notoginseng* saponin inhibits balloon injury-induced neointimal hyperplasia in rat carotid artery models by suppressing pERK/p38 MAPK pathways

**DOI:** 10.1590/1414-431X20199085

**Published:** 2019-12-20

**Authors:** Zheng Yang, Hui Zhang, Ming An, Mengni Bian, Miao Song, Xiaohua Guo, Quanli Liu, Min Qiu

**Affiliations:** 1 Baotou Medical College, Baotou, Inner Mongolia, China; 2 Second Department of Cardiovascular Diseases, First Affiliated Hospital of Baotou Medical College, Baotou, Inner Mongolia, China

**Keywords:** Total *Panax notoginseng* saponin, Neointimal hyperplasia, Vascular injury, Phosphorylated extracellular signal-regulated kinase1/2, p38 mitogen-activated protein kinase

## Abstract

Total *Panax notoginseng* saponin (TPNS) is the main bioactivity compound derived from the roots and rhizomes of *Panax notoginseng* (Burk.) F.H. Chen. The aim of this study was to investigate the effectiveness of TPNS in treating vascular neointimal hyperplasia in rats and its mechanisms. Male Sprague-Dawley rats were randomly divided into five groups, sham (control), injury, and low, medium, and high dose TPNS (5, 10, and 20 mg/kg). An *in vivo* 2F Fogarty balloon-induced carotid artery injury model was established in rats. TPNS significantly and dose-dependently reduced balloon injury-induced neointimal area (NIA) (P<0.001, for all doses) and NIA/media area (MA) (P<0.030, for all doses) in the carotid artery of rats, and PCNA expression (P<0.001, all). The mRNA expression of smooth muscle (SM) α-actin was significantly increased in all TPNS groups (P<0.005, for all doses) and the protein expression was significantly increased in the medium (P=0.006) and high dose TPNS (P=0.002) groups compared to the injury group. All the TPNS doses significantly decreased the mRNA expression of c-*fos* (P<0.001). The medium and high dose TPNS groups significantly suppressed the upregulation of pERK1/2 protein in the NIA (P<0.025) and MA (P<0.004). TPNS dose-dependently inhibited balloon injury-induced activation of pERK/p38MAPK signaling in the carotid artery. TPNS could be a promising agent in inhibiting cell proliferation following vascular injuries.

## Introduction

Vascular smooth muscle cells (VSMCs) are a major structural component of the vessel wall and play a key role in maintaining vascular structure. It is currently thought that intact endothelium is a key inhibitor of VSMC proliferation (1). However, under pathophysiological conditions such as coronary heart disease (CHD), hypertension (HTN), diabetes, and percutaneous coronary intervention (PCI), the endothelium may be damaged. Vascular injury provokes neointimal hyperplasia and vessel remodeling by inducing aberrant VSMC proliferation and migration, which further reduces blood flow and aggravates vascular luminal narrowing and may result in cardiovascular disease (2,3). Therefore, suppressing the proliferation and migration of VSMC could play an important role in preventing the pathological process of neointimal hyperplasia and might become a novel therapeutic strategy. Currently, there is a lack of effective drugs for controlling neointimal hyperplasia following vascular injury. A drug with an effective anti-neointimal function could be widely applied in clinical practice for the prevention and treatment of CHD and HTN, and other relevant diseases.

Total *Panax notoginseng* saponin (TPNS), extracted from the roots and rhizomes of *Panax notoginseng* (Burk.) F.H. Chen, is a widely used traditional Chinese medicine in China and other Asian countries (4,5). It is composed of ginsenosides Rg1, Rb1, and Rd, and notoginsenoside R1 (6,7). TPNS exhibits various pharmacological effects, which have been popularly used to treat cerebral infarction and ischemia, CHD, atherosclerosis, and trauma, and as anti-inflammation, anti-apoptosis, anti-thromboembolism, anti-coagulation, anti-hyperglycemia, and anti-hyperlipidemia drugs (8–10). Wang et al. (11) also demonstrated that *Panax notoginseng* saponin (PNS) exhibits significant cardio-protective effects *in vivo*. Furthermore, it has been reported that PNS protects cardiomyocytes from ischemia-induced apoptosis via activating the (phosphatidylinositol 3-kinase/protein kinase B) PI3K/Akt signaling pathway (12). PNS is also known to inhibit vascular intimal hyperplasia and VSMC proliferation (13–16). Our previous studies demonstrated that TPNS inhibited platelet-derived growth factor-BB (PDGF-BB)-induced artery smooth muscle cell proliferation *in vitro* by preventing the transformation of the G0/G1phase cells to S phase cells (17). Moreover, Cui et al. demonstrated that Akt, extracellular-signal-regulated kinase (ERK), and p38 mitogen-activated protein kinase (MAPK) signaling pathways can target PDGF, which is known to promote cell proliferation and migration (18). These data indicated that there is a correlation between the therapeutic potential of TPNS and the MAPK signaling pathway. However, whether TPNS inhibits VSMC proliferation following balloon induced-injury in rats awaits further investigation. Therefore, this study investigated the anti-neointimal effect of TPNS on hyperplasia using balloon-induced carotid artery injury rat model and its underlying mechanisms.

## Material and Methods

### Animals

Male Sprague-Dawley rats weighing approximately 280 g were obtained from the Department of Laboratory Animal Science of Peking Health Science University (China) and housed under a 12-h light/dark cycle with free access to food and water. The animals were anesthetized via intraperitoneal (*ip*) injection of 3% sodium pentobarbital at a dose of 1 mL/kg body weight. All experimental protocols related to use and treatment of animals were approved by the Animal Ethics Committee of Baotou Medical College.

### Chemicals

TPNS was purchased from the National Institutes for Food and Drug Control (China), batch number 110870-201002, purity 75.7%; it was composed of R1 (6.9%), Rb1 (29.7%), Re (3.8%), Rd (7.3%), and Rg1 (28.0%). Before administration, TPNS was suspended in saline.

### Experiment protocol and rat carotid artery balloon injury model

The animals were randomly divided into 5 groups of n=10/group: sham (control) group (operated without inducing injury), injury group (operated, injured, treated with distilled water), and 3 TPNS groups (operated, injured, and treated with 5, 10, and 20 mg/kg (low, medium, and high dose) for 14 days). To induce injury, a balloon catheter (2F Fogarty; Edwards Lifesciences Co., USA) was inserted through the left external carotid artery (ECA) into the common carotid artery (CCA), and distended with 0.1 mL physiological saline 3 times. The ECA was ligated after removal of the catheter and the wound was closed. After the operation, the animals were intramuscularly treated with antibiotics (0.12×10^6^ IU penicillin G benzathine, North China Pharmaceutical Co., Ltd., China) to prevent infection. In the sham group, the left CCA and ECA of the rats were exposed and ligated as above, but the catheter was not inserted into the vessels. All groups were administered distilled water or TPNS orally from the day following the operation for 2 weeks continuously. After the last administration, the animals were maintained for 24 h and then sacrificed via *ip* injection of 3% sodium pentobarbital. The left CCA was extracted for morphological examination, immunohistochemical staining, and western blot analysis according to our previously described methods (19).

### Morphologic observations

After routine H&E staining, the slices were observed under 100× magnification with Leica light microscope (Germany) and the neointimal area (NIA) and media area (MA) were examined using Image-Pro Plus image analysis system (a computer-assisted morphometry manipulation system; Media Cybernetics, Inc. USA) to obtain the NIA/MA ratio. NIA/MA ratio was used to indicate the degree of intimal hyperplasia. The neointimal layer is defined as the region between the vessel lumen and the internal elastic fibers within the vascular wall, and the media layer is defined as the region between the internal and external elastic fibers (20).

### Immunohistochemistry

Protein expressions of the proliferating cell nuclear antigen (PCNA) was detected by immunohistochemical methods. The PCNA mouse anti-rat monoclonal antibody was purchased from Wuhan Booster Biological Engineering Company (China). The secondary antibody used was biotinylated affinity associated rabbit immunoglobulin G (IgG) to mouse IgG. Diaminobenzidine (DAB) was utilized as chromogen and the slides were counter-stained with hematoxylin. At the same time, the slides of the negative control group were processed with omission of the primary antibodies to assess the presence of PCNA. The percentage of PCNA-positive cells was calculated as: PCNA-positive cells (%) = [(number of PCNA-positive cells) / total number of cell nuclei] ×100%.

The normal cell number and PCNA-positive cell number were counted in 5 fields of every section under the light microscope. The expression of SM α-actin and pERK1/2 (Boster Biological Technology Co. Ltd, China) were quantitatively measured with integral absorbance/area (mean density), and separately counted in the intima and media. The mean density represents the expression level.

### RNA extraction and real time RT-PCR

Total RNA was extracted from the VSMC using TRIzol reagent (Invitrogen, USA) according to the manufacturer's instructions. Synthesis of cDNA was performed using the total RNA by the reverse transcription system (Promega, USA) and oligo (dT) primers (Thermo Fisher Scientific, Inc., USA) according to the manufacturer's instructions. Real time reverse transcription polymerase chain reaction (RT-PCR) was performed using the Applied Biosystems 7900 real time RT-PCR System (Bio-RAD, USA), with SYBR Green PCR Master Mix (Promega). The primers were synthesized by TaKaRa Biological Engineering Company (China), as follows: β-actin forward: 5′-GGAGATTACTGCCCTGGCTCCTA-3′; β-actin reverse: 5′GACTCATCGTACTCCTGCTTGCTG-3′; c-*fos* forward: 5′-TCAATCCCTCCCTCCTTTACAC-3′; c-*fos* reverse: 5′-GTAGGATTTCGGGGATGGTTC-3′; SM α-actin forward: 5′-AGGGCTGTTTTCCCATCCAT-3′; SM α-actin reverse: 5′-GCTGTCCTTTTGGCCCATT-3′. The reaction conditions were: 95°C for 2 min, 1 cycle; 95°C for 15 s, 60°C for 1 min, 45 cycles. The threshold cycle (Ct) values of target genes were normalized to β-actin and are reported as relative to control taking control as 100%.

### Western blot assay

Western blotting was performed as previously described (21). The balloon-injured carotid artery samples were cut into pieces and put into 1-mL radio-immunoprecipitation assay (RIPA) lysing buffer (Beyotime Biotechnology, China). The lysing was ended with 1 mM phenylmethane sulfonyl fluoride (PMSF) (Beyotime Biotechnology). Then, the samples were homogenized and centrifuged (4°C, 5000 *g*, 15 min), and the supernatants were collected. The total protein in the supernatants was quantified by bicinchoninic acid (BCA) protein assay kit (Beyotime Biotechnology). TBS (1× Tris-buffered saline) loading buffer was added according to the proportion of 4:1 by volume (about 25 μL 1× TBS loading buffer was added into each tubule of protein) and boiled for 5 min. The final protein sample was obtained after blending. Total protein (100 μg) was subjected to electrophoresis on 4-12% gradient sodium dodecyl sulfate-polyacrylamide gel electrophoresis (SDS-PAGE), followed by electrophoretic transfer to poly-vinylidene fluoride (PVDF) membranes at 40 V for 4 h at 4°C. The membranes were blocked with 5% defatted milk in 15 mM Tris-HCl, pH 7.4, 150 mM NaCl, and 0.1% Tween 20 (Tris-buffered saline with Tween 20, TBST) for 1 h at room temperature (RT) followed by incubation with primary antibodies pERK (A01992-2) and p38 MAPK (BM4439) purchased from Boster Biological Technology Co., Ltd. (China), and incubated in 1% defatted milk in TBST overnight at 4°C, followed by secondary antibody at RT for 2 h. The image was scanned and the band densities were quantified using Quantity One 1D analysis software v4.52 (BioRad, USA). GAPDH was used to normalize protein loading.

### Statistical analysis

All data are reported as means±SE, and were subjected to one-way analysis of variance (ANOVA) and Tukey's HSD *post-hoc* test. SPSS 19.0 software (IBM, USA) was used for statistical analysis. Fisher's least square difference (LSD) test was employed to determine significance. The criterion for significance was set at P<0.05.

## Results

### TPNS inhibited intimal hyperplasia

**Figure 1:**
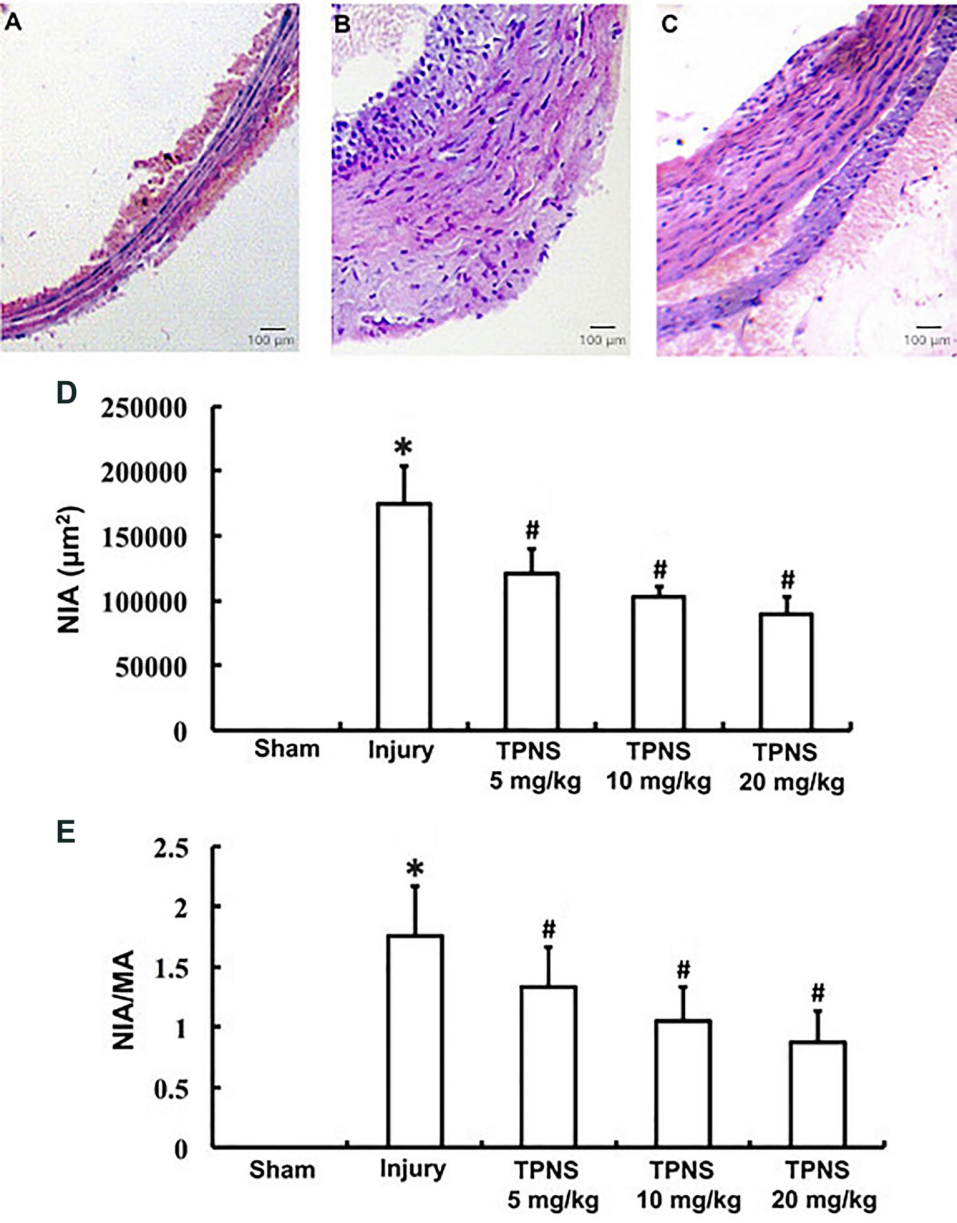
Effect of total *Panax notoginseng* saponin (TPNS) on neointimal hyperplasia induced by balloon-injury. **A**, **B**, and **C** demonstrate neointimal hyperplasia sections (H&E 100×, bar: 100 μm) in the sham group, injury group, and high dose (20 mg·kg^-1^·day^-1^) TPNS-treatment group, respectively. **D** and **E,** data obtained from the Image-Pro Plus analysis system showing mean values of neointimal area (NIA) and NIA/media area (MA) ratio, respectively, after intervention in the sham group, injury group, and low, medium, and high dose TPNS-treatment groups (5, 10, and 20 mg·kg^-1^·day^-1^). Data are reported as means±SE of 10 rats per group. *P<0.05 compared to Sham; ^#^P<0.05 compared to injury (ANOVA and Tukey's HSD *post hoc* test).

Results of the pathological examinations showed that no neointimal hyperplasia occurred in the sham group (Figure 1A). In the injury group, however, the neointima was significantly thickened (Figure 1B). Compared to the injury group, TPNS significantly alleviated the thickening of NIA, but it remained significantly greater than the sham group (Figure 1C). The TPNS-induced decrease in NIA was dose-dependent with an approximate 50% restoration of injuries in subjects with high dose TPNS (Figure 1D). A similar pattern was noted in NIA/MA. As shown in Figure 1D and E, the NIA and NIA/MA was 0 in the sham group and was significantly different than the injury group (NIA, P=0.000; NIA/MA, P=0.000) and all the TPNS groups (NIA, P=0.000; NIA/MA, P=0.000, all). The NIA and NIA/MA were reduced by approximately 50% in the high dose TPNS group compared to the injury group (NIA, P=0.000; NIA/MA, P=0.000).

### Influence of TPNS on PCNA staining and protein expression levels

**Figure 2:**
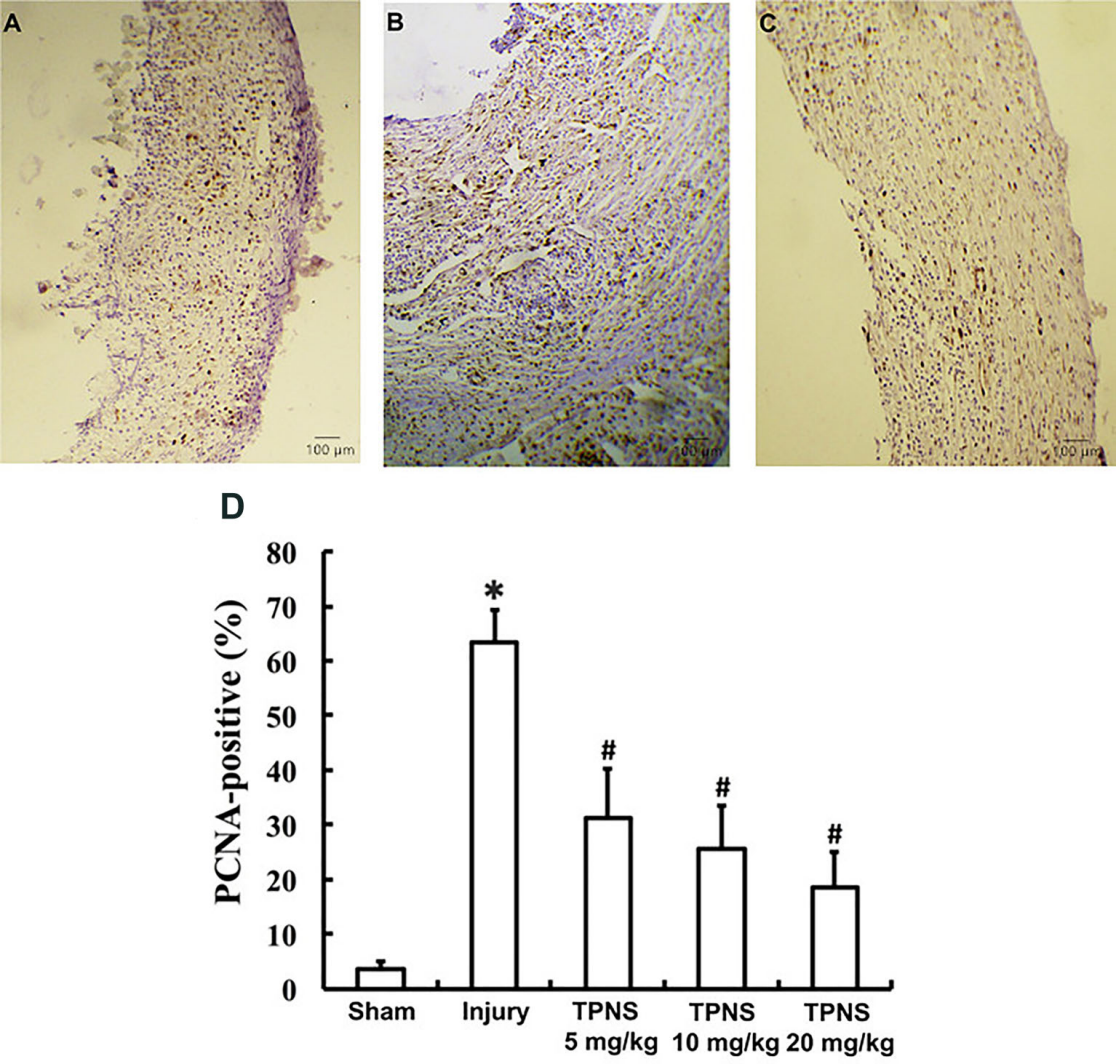
Inhibitory effect of total *Panax notoginseng* saponin (TPNS) on the percentage of PCNA-positive cells in the neointima of balloon-injured carotid arteries of rats. Representative sections (original magnification 100×, bar: 100 μm) of carotid arteries with PCNA immunohistochemistry staining show almost no PCNA-positive cells in the sham group vascular walls (**A**), a large number of PCNA-positive cells in balloon injured vascular walls (**B**), and a decreased number of PCNA-positive cells in the high dose (20 mg·kg^-1^·day^-1^) TPNS-treatment group (**C**). **D**, Data taken from the image manipulation system show changes of PCNA-positive cell percentage after TPNS administration at different doses for 14 days (percentage of PCNA-positive cell count per total cell count). Data are reported as means±SE of 10 rats per group. *P<0.05 compared to Sham; ^#^P<0.05 compared to injury (ANOVA and Tukey's HSD *post hoc* test).

As shown in Figure 2A, there were few PCNA-positive cells (brown nuclei) in the CCA wall in the sham group, but were significantly increased in the injury group (Figure 2B). Compared to the injury group, the TPNS groups exhibited significantly reduced numbers of PCNA-positive cells (Figure 2C). The data in Figure 2D show the percentage of PCNA-positive cells. Compared to the sham group, there was an increase of approximately 65% in the number of PCNA-positive cells following vascular injury (P=0.000). Compared to the injury group, TPNS produced a marked reduction to 30, 25, and 20% in groups dosed 5, 10, and 20 mg/kg, respectively (P=0.000, all).

### Effects of TPNS on SM α-actin protein expressions

**Figure 3:**
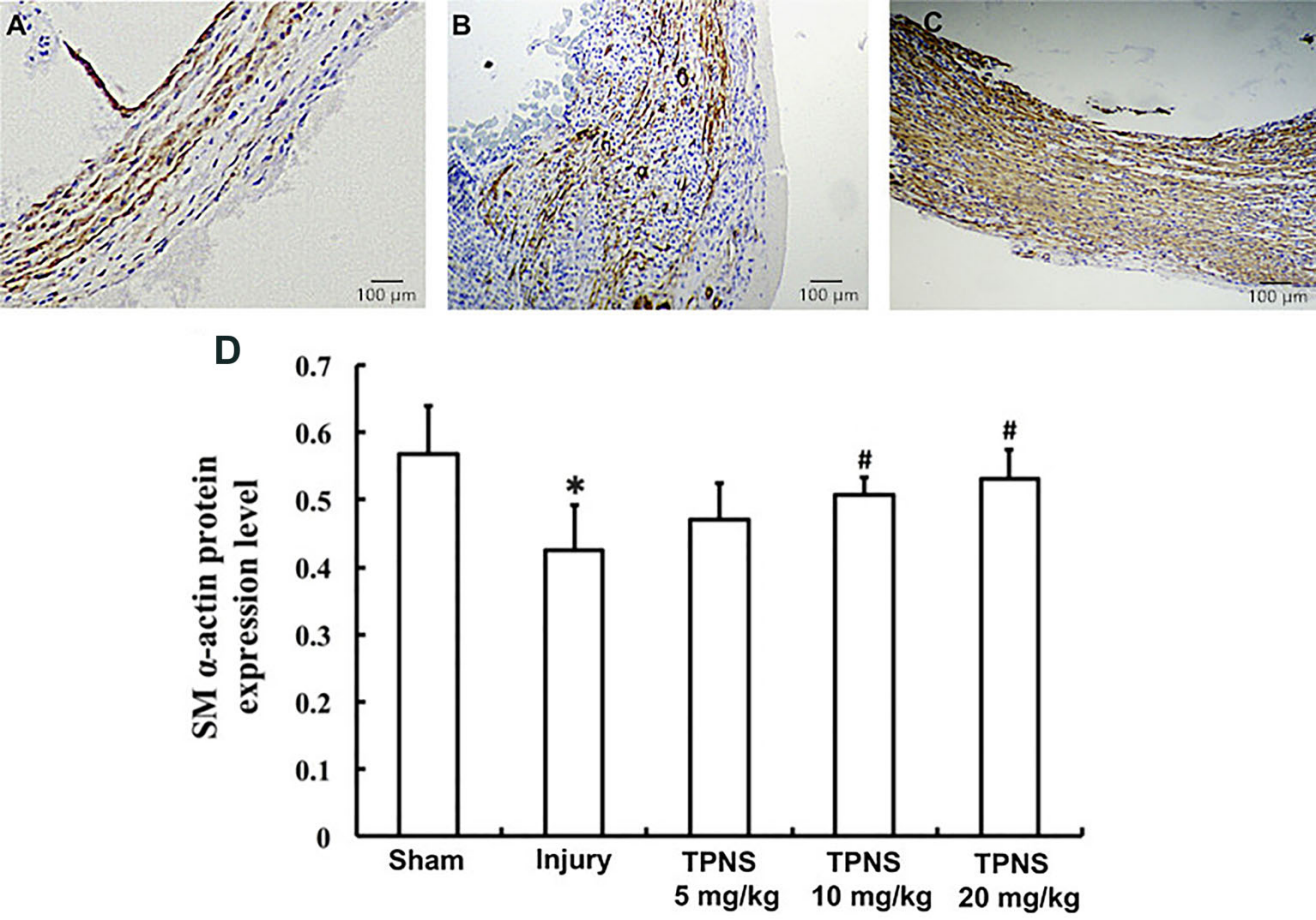
Effects of total *Panax notoginseng* saponin (TPNS) on smooth muscle (SM) α-actin protein in balloon-injured carotid artery walls. **A**, **B**, and **C** show representative sections (original magnification 100×, bar: 100 μm) of carotid arteries with SM α-actin protein immunohistochemistry staining in the sham group, injury group, and high dose (20 mg·kg^-1^·day^-1^) TPNS-treatment group, respectively. The total SM α-actin protein expression levels in the combined neointimal area (NIA) and media area (MA) layers in the sham group, injury group, and low, medium, and high TPNS-treatment groups (5, 10, and 20 mg·kg^-1^·day^-1^) are quantitatively shown in **D**. Data are reported as means±SE of 10 rats per group. *P<0.05 compared to Sham; ^#^P<0.05 compared to injury (ANOVA and Tukey's HSD *post hoc* test).

As illustrated in Figure 3, positive SM α-actin staining in the sham group was considerable (Figure 3A). In the injury group, positive staining was rare in the neointima (Figure 3B). High dose TPNS (20 mg·kg^-1^·day^-1^) treatment significantly elevated the occurrence of SM α-actin positive staining (Figure 3C). The data in Figure 3D show the total SM α-actin protein expression levels in combined NIA and MA layers. The TPNS-induced increase in SM α-actin protein expression levels was dose-dependent. The increments in the medium and high dose TPNS groups were significant compared to the injury group (P=0.006, P=0.002, respectively). The group treated with high dose TPNS demonstrated restoration of SM α-actin protein expression to levels similar to the sham group (P=0.080) (Figure 3D).

### Influence of TPNS on mRNA expression of oncogene c-*fos* and SM α-actin

**Figure 4:**
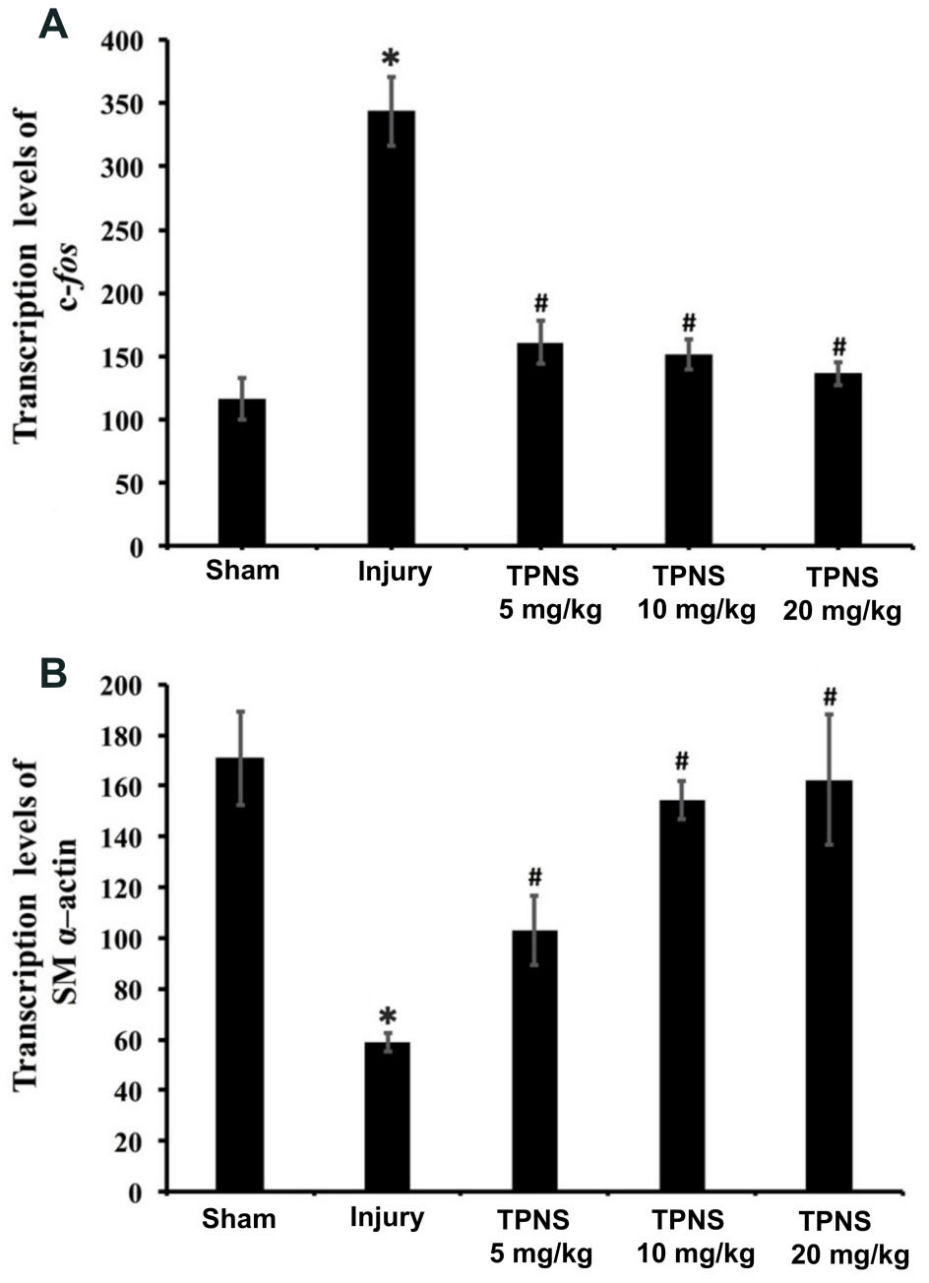
Effects of total *Panax notoginseng* saponin (TPNS) on c-*fos* and smooth muscle (SM) α-actin mRNA expression in injured arteries. The total expression levels of c-*fos* and SM α-actin mRNA in the artery walls in the sham group, injury group, low, medium, and high dose TPNS-treatment groups (5, 10, and 20 mg·kg^-1^·day^-1^) are shown in **A** and **B**, respectively. Data are reported as means±SE of 10 rats per group. *P<0.05 compared to Sham; ^#^P<0.05 compared to injury (ANOVA and Tukey's HSD *post hoc* test).

Compared to the sham group, the mRNA expression level of c-*fos* was elevated in the injury group (P=0.002). After TPNS treatment, there was a significant decrease in c-fos mRNA expression levels (P=0.001, P=0.000, P=0.000 for low, medium, and high doses, respectively). The data also demonstrated that the 3 TPNS doses produced an almost equal effect indicating a dose-independent response (Figure 4A). In contrast, the data in Figure 4B indicated a significant decrease in SM α-actin transcription levels in the injury group compared to the sham group (P=0.000). All 3 TPNS doses resulted in significant upregulation of SM α-actin mRNA expression in the injured CCA wall compared with the injury group (P=0.005, P=0.000, P=0.002 for low, medium, and high doses, respectively), which was consistent with the results of the immunohistochemistry staining assay.

### Effects of TPNS on pERK/p38 MAPK protein expression

**Figure 5:**
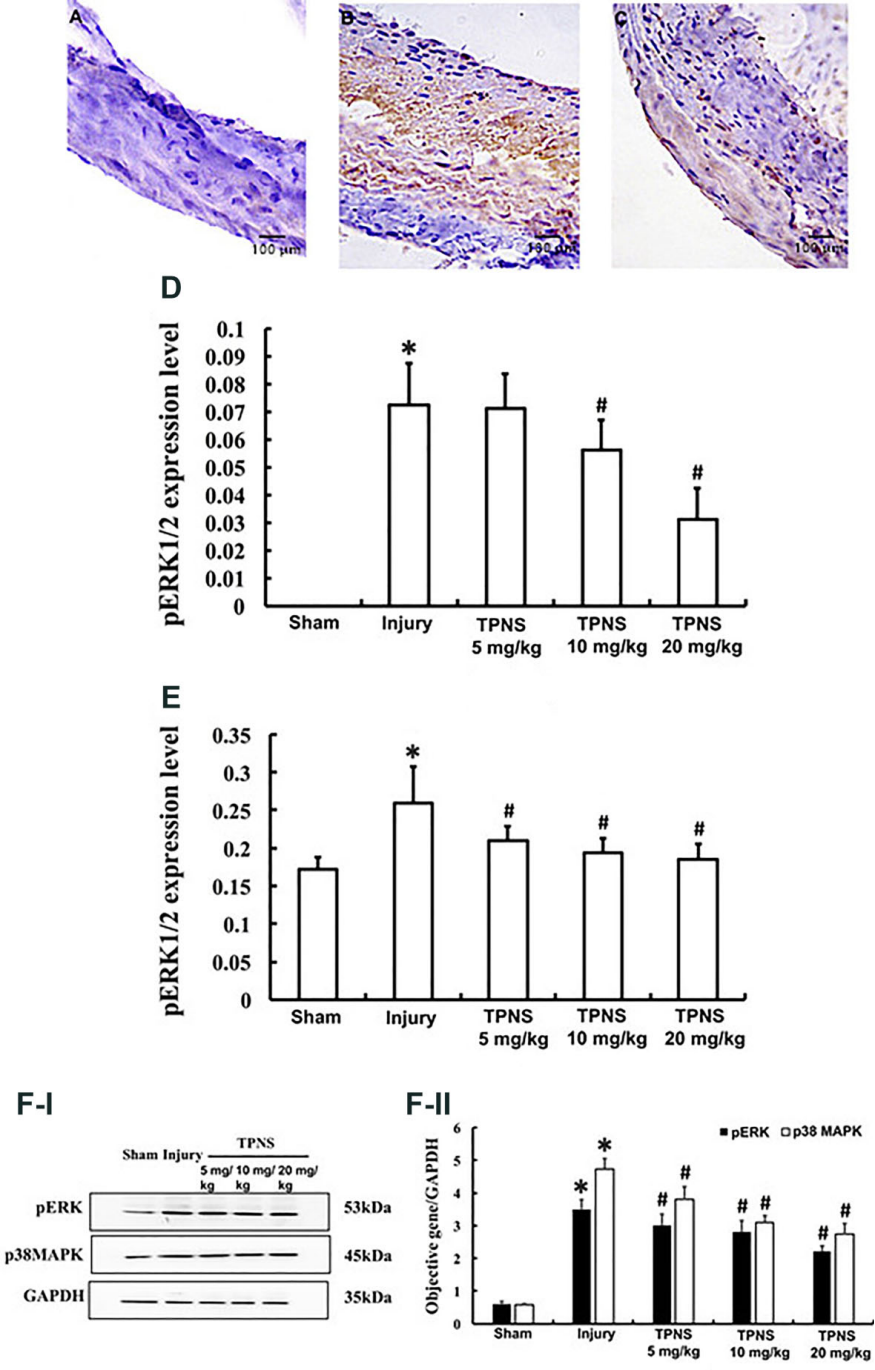
Effects of total *Panax notoginseng* saponin (TPNS) on the pERK/p38 MAPK signaling pathway in the neointima of balloon-injured carotid arteries of rats. **A**, **B**, and **C** show the area of positive pERK immunohistochemistry staining in the sham group, injury group, and high dose (20 mg·kg^-1^·day^-1^) TPNS treatment group, respectively. **D** and **E** show the levels of pERK 1/2 expression in the neointimal area and media area, respectively, in the sham group, injury group, and TPNS-treatment groups. **F**-**I** shows the protein bands of pERK, p38 MAPK, and GAPDH, and **F**-**II** shows the objective gene/GAPDH ratio in the sham group, injury group, and TPNS-treatment groups. Data are reported as means±SE of 10 rats per group. *P<0.05 compared to Sham; ^#^P<0.05 compared to injury (ANOVA and Tukey's HSD *post hoc* test).

There was a significantly elevated expression level of pERK1/2 protein in the injury group (Figure 5B) compared with the sham group (Figure 5A). Treatment with TPNS at doses of 10 and 20 mg/kg (Figure 5C) significantly suppressed the upregulation of pERK1/2 protein levels in both the NIA (P=0.024, P=0.000, respectively) and MA layers (P=0.003, P=0.001, respectively) (Figure 5D and E). Consistent results were observed in the NIA and MA layers (Figure 5D and E), whereas TPNS at 5 mg/kg exerted no marked effect on the pERK1/2 protein level in the NIA layer (P=0.858) (Figure 5D).

As expected, the results presented in Figure 5F indicated that balloon induced-injuries in the carotid artery could trigger significant elevation of pERK and p38 MAPK expression compared with the sham group (P=0.000, both). Likewise, TPNS could inhibit the increase of pERK and p38 MAPK protein expression levels, and the data suggested that TPNS was able to concentration-dependently inhibit balloon injury-induced activation of pERK/p38 MAPK signaling in the carotid artery (pERK, P=0.017, P=0.010, P=0.001; p38 MAPK, P=0.013, P=0.003, P=0.000, for low, medium, and high doses, respectively) (Figure 5F).

## Discussion

Previous studies have demonstrated that, following vascular injuries, blood vessels are affected by pathological factors and cell proliferation is initiated. The proliferation and migration of VSMC from the medial to the intima layer of the artery is a key early event and the main pathological process of vascular stenosis caused by neointima formation (22–24). Vascular stenosis may induce insufficient perfusion of tissues and organs. If stenosis is further aggravated, it will cause ischemia or even necrosis of the corresponding tissues. Taking PCI as an example, although drug-eluting stents have been widely used, excessive proliferation and migration of VSMC caused by stent implantation eventually leads to neointimal hyperplasia and stent restenosis (25,26). Therefore, inhibition of VSMC proliferation to prevent formation of neointima is considered a promising strategy to alleviate the narrowing of injured arteries. Our results showed that the neointima was significantly thickened following arterial injury. Treatment with TPNS for 14 days on arterial models with balloon-induced injury significantly counteracted the increase of NA and NA/NIA, suggesting that TPNS produced a protective effect on neointimal hyperplasia following balloon injury.

PCNA, an accessory protein of DNA polymerase δ, is a nuclear antigen which is directly involved in DNA synthesis (27), represents cell division and proliferation, and plays a key role in controlling cell cycle progression (28,29). When cells begin to proliferate, especially in the proliferative S phase, PCNA is significantly activated and up-regulated. If the PCNA gene expression is repressed, proliferation and migration of VSMC can be inhibited (30). Our previous research showed that TPNS markedly decreased the percentage of S phase cells and increased the percentage of G0/G1 phase cells in the cell population. In this study, we found that the percentage of positive PCNA staining was significantly elevated in the NIA and MA layers following arterial injury. In agreement with the morphological observations, a marked reduction in the percentage of PCNA-positive cells was observed following treatment with TPNS, indicating that this compound effectively alleviated intimal hyperplasia by inhibiting VSMC proliferation via blocking the cell cycle progress.

Next, we determined the inhibitory effects of TPNS on neointimal hyperplasia following arterial injury. There are two phenotypes for VSMC, contractile (differentiated) and synthetic (proliferative or dedifferentiated). Under normal circumstances, VSMC is highly specialized to a differentiated and quiescent contractile phenotype, which exhibits elevated levels of contractile proteins such as SM α-actin. In response to vascular impairment, however, VSMC switches to a dedifferentiated and proliferative synthetic phenotype with decreased levels of contractile proteins (31,32). In other words, SM α-actin, a key contractile marker, is concomitantly reduced during the cell proliferation process. Neointima formation resulting from vascular endothelial injury is also characterized by a reduction of SM α-actin protein and mRNA expression following balloon injury, and we observed higher SM α-actin expression levels following administration of TPNS. These data reaffirmed that TPNS has the ability to alleviate vascular injury-induced VSMC proliferation and neointimal hyperplasia, which is associated with phenotypic switching.

VSMC phenotypic switching have been associated with the MAPK pathway and Akt signaling (33,34). The maintenance of a contractile phenotype in VSMC was shown to be dependent on the Akt pathway, whereas activation of the ERK and p38 MAPK pathways induced proliferation (35–37), thus indicating that the VSMC phenotype is determined by a balance between these pathways. MAPK is a group of protein kinases widely existing in cells with serine/threonine bi-phosphorylated protein kinase. The family mainly contains extracellular adjusting kinase (extracellularly regulated kinase, ERK), c-Jun N-terminal kinase (JNK), and p38 (38). ERK and p38 MAPK have been associated with VSMC phenotype switching, but JNK has not (39). Under some pathological conditions, mitosis of VSMC is initiated by MAPK protein kinase phosphorylation, which activates mitogen-activated protein kinase kinase (MEK), ERK1/2, and p38 MAPK, and promotes high expression of early gene c-*fos* by phosphorylating downstream substrate molecules to promote cell proliferation (40). Therefore, blocking the MAPK/ERK signaling pathways inhibits VSMC proliferation. In this study, balloon injury induced pERK and p38 MAPK activation in the carotid artery. Treatment with TPNS strongly inhibited or tended to reduce the pERK and p38 MAPK protein expression induced by vascular injury. In agreement with the changes of pERK and p38 MAPK, TPNS also significantly decreased the mRNA expression of c-*fos*.

Neointimal formation after vascular injury is common in CHD, HTN, diabetes, PCI, and other circulatory system diseases. The cause is mainly due to increased blood pressure, raised blood sugar level, or mechanical injury resulting in neointimal formation leading to luminal narrowing. The incidence of CHD, HTN, and atherosclerosis is increasing each year and there is a growing trend in younger patients.

Drug-eluting stents have been widely used in PCI treatment, however, stenting can cause vascular endothelial injury and lead to overproliferation of VSMC, migration, and neointimal formation. This causes stent restenosis and even complete occlusion and remains the key link in surgical failure. It is also an important cause of serious cardiovascular events. High incidence of CHD, HTN, and diabetes can lead to an increased incidence of cell proliferation and neointimal formation following vascular injury, resulting in vascular stenosis, which affects blood flow and causes ischemic symptoms, and the condition is indeed serious.

However, there are currently no clinically effective methods or drugs for controlling proliferation and migration of VSMC. A drug with definite anti-VSMC proliferation could be widely used in clinical practice for the prevention and treatment of neointimal formation in CHD, HTN, diabetes, and PCI, which can lead to vascular stenosis. This is also a research hotspot in the cardiovascular field.

Our study found that TPNS can inhibit neointimal hyperplasia following vascular injury, and it may have potential as a new therapeutic strategy in the prevention and treatment of vascular stenosis due to neointimal hyperplasia following vascular injury in CHD, HTN, diabetes, PCI, and other circulatory system diseases. The limitation of this study is that a rat model was used to simulate human pathology. Further study on the long-term therapeutic effects and clinical trials would be helpful.

We conclude that our results suggested that TPNS exerted a protective role against neointimal hyperplasia after balloon injury in rats by suppressing VSMC proliferation, which involves inhibition of pERK/p38 MAPK signaling pathway. These findings indicated that TPNS may be considered a promising agent in inhibiting cell proliferation following vascular injuries and relevant lesions.
